# When does the female bias arise? Insights from the sex determination cascade of a flea beetle with a strongly skewed sex ratio

**DOI:** 10.1007/s10142-023-01023-1

**Published:** 2023-03-31

**Authors:** Kim Rohlfing, Lennart Yue, Sebastian Franke, Cen Zeng, Lars Podsiadlowski, Susanne Dobler

**Affiliations:** 1 Institute of Animal Cell and Systems Biology, Universität Hamburg, Martin-Luther-King-Platz 3, D-20146 Hamburg, Germany; 2 Leibniz Institute for the Analysis of Biodiversity Change, Museum Koenig, Bonn, Germany

**Keywords:** *dsx*, *tra*, *tra2*, *Altica lythri*, *Wolbachia*, Chrysomelidae, Sex determination, Female bias

## Abstract

Reproduction-manipulating bacteria like *Wolbachia* can shift sex ratios in insects towards females, but skewed sex ratios may also arise from genetic conflicts. The flea beetle *Altica lythri* harbors three main mtDNA strains that are coupled to three different *Wolbachia* infections. Depending on the mtDNA types, the females produce either offspring with a balanced sex ratio or exclusively daughters. To obtain markers that can monitor when sex bias arises in the beetle’s ontogeny, we elucidated the sex determination cascade of *A. lythri.* We established a RT-PCR method based on length variants of *dsx* (doublesex) transcripts to determine the sex of morphologically indistinguishable eggs and larvae. In females of one mtDNA type (HT1/HT1*) known to produce only daughters, male offspring were already missing at the egg stage while for females of another type (HT2), the *dsx* splice variants revealed a balanced sex ratio among eggs and larvae. Our data suggest that the sex determination cascade in *A. lythri* is initiated by maternally transmitted female-specific *tra* (transformer) mRNA as primary signal. This *tra* mRNA seems to be involved in a positive feedback loop that maintains the production of the female splice variant, as known for female offspring in *Tribolium castaneum*. The translation of the maternally transmitted female *tra* mRNA must be inhibited in male offspring, but the underlying primary genetic signal remains to be identified. We discuss which differences between the mtDNA types can influence sex determination and lead to the skewed sex ratio of HT1.

**Supplementary Information:**

The online version contains supplementary material available at 10.1007/s10142-023-01023-1.

## Introduction

Sex determination cascades trigger the developmental mechanism that shapes embryos into males or females (Sanchez 2008). These mechanisms can provide crucial insights into our understanding of population structures and the evolution of sex. We here elucidate the sex determination cascade of a flea beetle species with strongly female-biased sex ratios, pervading *Wolbachia* infections and a complex hybridogenetic origin (Jäckel et al. 2013) to gain insights into when and how female bias develops.

Insects harbor various mechanisms for sex determination (Gempe and Beye 2011; Marín and Baker 1998; Sanchez 2008), including genetic sex determination, paternal genome elimination, and haplodiploidy (Bachtrog et al. 2014; Blackmon et al. 2017; Erickson and Quintero 2007; Sanchez 2008). This diversity of mechanisms originates from the variability in the primary sex determination cascade trigger. Consequently, even closely related species have evolved distinct primary sex determination signals (Bopp et al. 2014; Sanchez 2008; Verhulst et al. 2010). Even in extensively studied insect model organisms like *Drosophila melanogaster* and *T. castaneum*, the signal that triggers the sex determination cascade is not fully understood. In contrast, the downstream, central sex determination pathway is well conserved between insect groups (Graham et al. 2003; Wilkins 1995). Following initiation, the conserved sex determination pathway, as observed in *T. castaneum*, begins with sex-specific splicing of the transformer (*Tra*) gene, which results in premature stop codons in the male version of transformer and, consequently, a truncated protein (Beukeboom et al. 2007). In the female, a functional Tra protein is formed that acts as an RNA splicing regulator to induce female-specific splicing of doublesex (*Dsx*) (Bopp et al. 2014; Sanchez 2008; Verhulst et al. 2010). In the male pathway, where the Tra protein is absent, *Dsx* is spliced into the male version *dsxm* (Cline and Meyer 1996). Dsx as a transcription factor is responsible for sex-specific traits, as male- and female-specific Dsx initiate the expression of different downstream genes and lead to the sex differences.

The determination of sex can further be influenced by selfish genetic elements and inherited bacterial endosymbionts, such as the α-proteobacterium *Wolbachia pipientis* (Cordaux et al. 2011; Hertig and Wolbach 1924). Like mitochondria, these bacteria are vertically transmitted from mother to offspring and are known to manipulate sex ratios. Infections can lead to male killing, feminization of genetic males, or induction of parthenogenesis (Cordaux et al. 2011; Werren et al. 2008). Similar to *Wolbachia*, mitochondria could increase their representation in a population by disfavoring males (Perlman et al. 2015). Although strong effects of mitochondrial types on male fitness have been detected (Frank and Hurst 1996; Innocenti et al. 2011; Ruiz-Pesini et al. 2000), a complete lack of males could so far not be connected to mitochondrial effects.

When deviations from the expected sex distribution of the offspring occur, many possibilities and time points exist at which sex ratio can be altered. This is especially so in hybrid species, which carry a higher potential for genetic conflicts (Franchini et al. 2018). The analysis of a hybrid species with obvious reproductive anomalies can therefore provide insight into the basic mechanisms of sex determination, the effects of hybridization on genetic conflicts, and possibly the influences of reproduction manipulating microorganisms.

*Altica lythri* (Coleoptera, Chrysomelidae) flea beetles show evidence of historic hybridization and unique reproductive anomalies that provide an ideal model for investigating how sex is influenced by genetic conflicts. The species is widespread throughout central Europe and notorious for its strongly female-biased sex ratio (Kangas and Rutanen 1993; Siede 1998). Ancient hybridization among *Altica* species and subsequent backcrossing resulted in the introgression of mitochondrial mtDNA, so that three major haplotypes (HT1, HT2, and HT3) can be found in this hybrid species today (Jäckel et al. 2013). The beetles are usually infected with different strains of *Wolbachia* bacteria (wLytA1, wLytA2, or wLytB) depending on their mitochondrial haplotype. Only a slight sequence variant of HT1, named HT1*, was found to be mostly uninfected. Intriguingly, beetles with HT1 and HT1* haplotype produce exclusively female offspring, whereas beetles with HT2 or HT3 haplotype show a more balanced sex ratio in their progeny (Jäckel et al. 2013). Depending on the proportion of the HT1 mtDNA haplotype in a population, the sex ratio can be shifted almost entirely towards females. The underlying mechanisms or critical points in time that are responsible for the exclusion of males are not known yet. Studies addressing this issue (Jäckel 2011) were hampered by the fact that the sex ratio could only be determined once the offspring reached the adult stage, since eggs and larvae of both sexes are morphologically indistinguishable.

The aim of this study was to describe and investigate the genetic sex determination cascade of *A. lythri* and determine at which timepoint the males disappear in the progeny of individuals with mtDNA HT1 and HT1*. The identification of the conserved elements of the sex determination cascade (transformer (*tra*), transformer 2 (*tra2*), and doublesex (*dsx*)) and their splice variants in both sexes enabled us to identify the sex and the sex ratio of eggs and larvae of HT1/HT1* and HT2 progeny. Knowing when the shifted sex ratio first occurs, we discuss the sex determination primary signal in *A. lythri* and the potential role of parasitic endosymbionts, like *Wolbachia*, or possibly an introgressed mtDNA in disfavoring phenotypic males.

## Materials and methods

### Beetle collection and captive breeding

*A. lythri* were collected during spring and summer 2018 and 2021 from populations in northern Germany in Güster (53°32′24.1″N 10°41′10.0″E), Büchen (53°28′42.4″N 10°37′56.2″E), Pevestorf (53°03′57.8″N 11°27′23.2″E) and in the Netherlands in Bergen (52°68′08.9″N 4°69′74.5″E). The beetles were sexed morphologically based on differences in the shape of their last abdominal sternites (Jäckel 2011), and their mtDNA haplotype was determined by PCR–RFLP (see below). The beetles were separated by population, sex, and haplotype and kept in a climatic chamber at 18 °C with 12-h light and 12-h darkness.

### Determination of mtDNA haplotype and Wolbachia infection status

To determine a priori the mtDNA haplotype of each beetle used in the experiments, DNA was extracted with the innuPREP Forensic Kit (Analytic Jena, Jena, Germany) from feces of live beetles kept in 1.5-ml reaction vials for 3 h, following the manufacturer’s protocol for animal tissues. *Wolbachia* infection was later determined from DNA extracted after freezing the insects. The mtDNA haplotypes and *Wolbachia* infection status were determined via PCR–RFLP as established in a previous study (Jäckel 2011). Briefly, *COI* (for mtDNA HT) and *wsp* (for *Wolbachia*) were amplified with specific oligonucleotides (supplementary table S1, supplementary material online). The PCR products were digested with *Hinf*I (Thermo Fisher) for *COI* and *Mae*I (Thermo Fisher) for *wsp* and separated on a 1.5% agarose gel. The fragment patterns could be assigned to specific mtDNA haplotypes or *Wolbachia* strains based on prior knowledge of their sequences and existing cut sites (Jäckel 2011).

### Sequence data collection

To identify the genes responsible for the sex determination cascade in *A. lythri*, transcriptomes of adults and larvae were generated. Total RNA from one *A. lythri* adult male (HT2) and three adult females (HT1, HT1*, HT2) from Büchen (Germany) and two HT2 larvae (one male and one female according to *dsx* PCR (see below)) were extracted using the RNeasy Plus Mini Kit (Qiagen, Hilden, Germany) according to the manufacturer’s instructions. Concentration and purity were checked on a NanoDrop 2.0 (Thermo Fisher). The transcriptome of each sample (about 4–5 μg RNA in 25 μl) was sequenced by a commercial service (StarSeq GmbH, Mainz, Germany) on an Illumina NextSeq2000 (2 × 150 nt). The quality of the RNAseq reads was controlled with FastQC. Trimmomatic, implemented in Trinity 2.4.0 (Grabherr et al. 2011; Haas et al. 2013), was used with default options to trim and filter the reads. Each dataset was separately assembled de novo with Trinity 2.4.0 with default parameters on the high-performance computing cluster “Hummel” at Universität Hamburg.

### Ortholog identification and sequence analysis

The *dsx, tra*, *tra2*, and *zld* transcript variants of *A. lythri* were identified using the tblastn algorithm (Altschul et al. 1990) on local blast databases in the de novo transcriptomes of male and female *A. lythri* (see above). Dsx protein sequences from *T. castaneum* (NP_001345539, NP_001345540, NP_001345541, NP_001345542), Tra protein sequences from *Cyclommatus metallifer finae* (Coleoptera, Lucanidae; BAV13588.1), and Zelda protein sequence of *T. castaneum *(XP_001812268.1) were used as queries. Multiple sequence alignments of the individual splice variants of each gene were performed by hand with Unipro UGENE v.1.29.0 (Golosova et al. 2014; Okonechnikov et al. 2012) and with the implemented MAFFT algorithm.

The exon–intron structures of *dsx*, *tra*, and *tra2* were obtained by mapping the transcriptomic sequences to a not yet fully polished genome of an *A. lythri* male (see below). Alignments of cDNA sequences with genomic DNA were performed online using splign (Kapustin et al. 2008).

Conserved protein domains were predicted by CD search (Marchler-Bauer et al. 2017) and comparison with different insect doublesex and transformer protein sequences in multiple alignments.

The genome assembly we used for gene structure analysis was based on Oxford nanopore long reads, polished with Illumina short reads (Novaseq 6000 platform, PE150). About 17 Gb of long reads were generated on the GridIon platform (Oxford Nanopore), with reads N50 of 8800. The assembly was done with flye 2.4 with the option “nano-raw” and an estimated genome size of 800 Mbp (Kolmogorov et al. 2019; Lin et al. 2016). Polishing for indel errors (a frequent problem with nanopore data) was done with 62 mio Illumina read pairs; after mapping the reads with BWA-MEM (Li and Durbin 2009, 2010), pilon was used to correct for indel and single nucleotide errors (Walker et al. 2014). The final polished genome assembly has a size of 844 Mb, is highly fragmented in 34,981 contigs, and has an N50 of 78 kbp.

### RNA extraction, cDNA synthesis, and reference plasmid cloning

To analyze differential gene expression during development, samples were collected and RNA extracted as follows. Female beetles with haplotypes HT1, HT1*, and HT2 were paired with one male (HT2) each. Every 24 h, the laid eggs or hatched larvae were collected and transferred to a new container to keep them separated by female and time point of collection until pupation of the larvae. The offspring of three females of each mtDNA haplotype were stored at − 80 °C until further processing: three pooled eggs for each female at days 7 and 14; one larva at days 1, 7, and 14; plus one pupa and one freshly eclosed beetle of each sex. Total RNA was isolated from the samples using RNAmagic (Bio-Budget, Krefeld, Germany) combined with the NucleoSpin® RNA XS kit (Macherey–Nagel) for qRT-PCR and the screening of sex in eggs and larvae (see below). The samples were frozen in liquid nitrogen and ground using a Teflon pestle; the resulting powder was mixed with 500 μl RNAmagic and vortexed thoroughly. After the addition of 100 μl chloroform and phase separation, the aqueous phase was further purified with the NucleoSpin® RNA XS kit including an on-column DNase digestion, according to the manufacturer’s instructions. RNA concentrations were measured by Qubit™ 3.0 (Thermo Fisher) with the Qubit™ RNA high sensitivity (HS) kit (Invitrogen), and the quality and integrity of the RNAs (50 ng) were checked by half-denaturing agarose gel electrophoresis. One hundred seventy-five nanogram RNA was reverse-transcribed with SuperScript™ III Reverse Transcriptase Kit (Invitrogen) with 2 μl oligo-(dT)_18_ primer in a total volume of 20 μl for 3 h at 50 °C. The cDNA was stored at − 20 °C.

To generate reference plasmids for standard curves, the genes of interest (*dsx*, *tra*, *tra2*, *zld*) were amplified with transcript-specific oligonucleotides (supplementary table S1, supplementary material online) and Taq polymerase (Invitrogen). The products were cloned into the pGEM-T vector (Thermo Fisher) in XL10Gold cells (Agilent Technologies, Waldbronn, Germany), purified via alkaline lysis, and sequenced by a commercial service (GATC, Köln, Germany). For the standard dilution series, the plasmids were linearized with ApaI (Thermo Fisher), dephosphorylated with calf intestinal alkaline phosphatase (Thermo Fisher), and purified using phenol/chloroform extraction and ethanol precipitation.

### Quantitative real-time reverse transcription polymerase chain reaction

The ontogenetic expression of *A. lythri* sex determination genes (*dsx*, *tra*, *tra2*) was analyzed by qRT-PCR performed on a StepOne system (Thermo Fisher) with a three-step amplification cycle (40 cycles: 95 °C for 15 s, 55 °C for 15 s, 72 °C for 1 min - detection at last step) using the Maxima SYBR Green/ROX qPCR Master Mix (Thermo Fisher). Melting curve analysis following the amplification cycles was used to evaluate the specificity of the amplification. Negative controls without cDNA were included on each plate.

Experiments were performed in triplicate in a volume of 10 μl with a final cDNA amount equivalent to 4.375 ng total RNA and 250 nmol of each intron-spanning oligonucleotide (supplementary table S1, supplementary material online). Standard curves with recombinant plasmids in tenfold serial dilutions were used to calculate the total mRNA copy number and primer efficiency. Each transcript sample was normalized to 1 μg RNA. Evaluation of the runs was done with the StepOne Software v2.2.2 (Thermo Fisher), and further calculations were made in GraphPad Prism 9. All information complies to the minimum information for publication of quantitative real-time PCR experiments (MIQE) guidelines (Bustin et al. 2009).

### Evaluation of the transfer of maternal mRNA

Potential transfer of maternal mRNA of the sex determination gene transformer was analyzed using eggs collected within 3 h after egg laying. The known maternally transferred mRNA of *zelda* was used as positive control. Eggs of female beetles were collected within 3 h after egg laying and frozen at − 80 °C in pools of 20 eggs obtained from different females separated by haplotype. The RNA of the pools was extracted with the NucleoSpin® RNA XS kit (Macherey–Nagel) according to the manufacturer’s instructions. RNA concentrations were measured by NanoDrop. cDNA synthesis was performed as described above with an RNA amount of 150 ng per reaction. *Traf* and *zld* were identified in the samples via qRT-PCR as described above in a two-step amplification protocol (40 cycles: 95 °C for 15 s, 60 °C for 1 min - detection at last step) with a final cDNA amount equivalent of 3.75 ng total RNA per well. RT negative controls (RT reaction mixes without reverse transcriptase) were also included to exclude amplification of DNA because the *zld* mRNA has no introns.

### Screening for sexes in eggs and larvae

The length differences of the sex-specific *dsx* transcripts were used to determine the sex of the morphologically indistinguishable eggs and larvae. Twenty-five HT1 eggs and 30 HT2 eggs (about 10 days old), as well as 42 HT1, 27 HT1*, and 45 HT2 larvae (about 2 weeks old), were sampled randomly from different breeding females. RNA extraction and cDNA synthesis were carried out from each individual egg or larva as described above. Oligonucleotides (dsx_f and dsxr, supplementary table S1, supplementary material online) that amplify all *dsx* splice variants were used to amplify the different *dsx* versions of males and females. Amplification was performed using Phusion Polymerase (Thermo Fisher) in 12.5 µl reactions [1 × HF buffer, 2 mM MgCl_2_, 200 µM of each dNTP, 500 µM of each oligonucleotide, 5% dimethyl sulfoxide, 1 µl cDNA] in 35 cycles (98 °C 3 min, 35 × (98 °C 10 s, 58 °C 30 s, 72 °C 30 s), 72 °C 7 min). The sizes of the generated products were analyzed on 1% agarose gels (Thermo Fisher) stained with ethidium bromide and visualized under UV light.

## Results

### Identification and characterization of the sex determination genes dsx, tra, and tra2 from transcriptomes of *A. lythri*

**Fig. 1:**
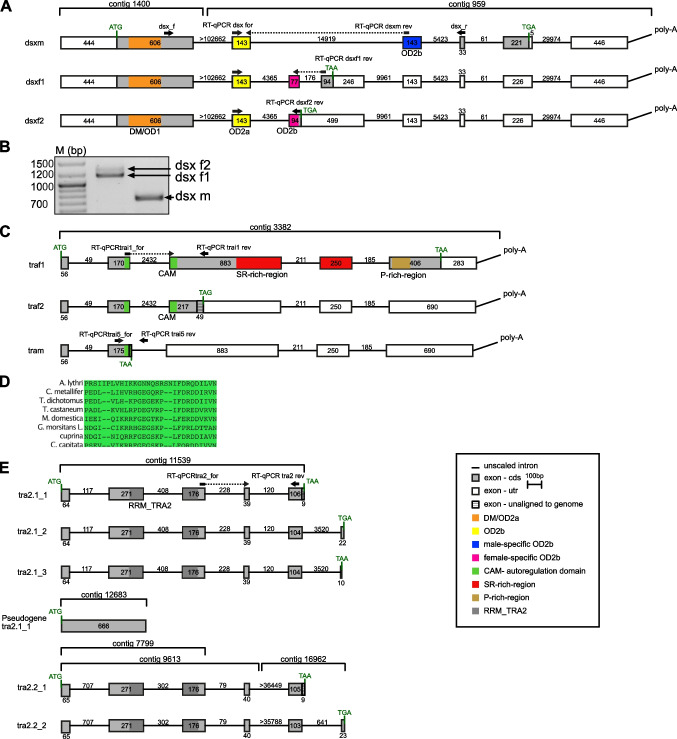
Schematic representations of the alignment of the splice variants of *Alydsx* (A), *Alytra* (C), and *Alytra2* (E) pre-mRNA. Exons are indicated with boxes (cds marked in grey); potential introns, detected by comparison with a draft genome, are indicated with lines, and numbers give lengths in bp. Color-coded/dark-grey shaded boxes show conserved domains. Primer positions are indicated as horizontal arrows. The primer pair dsx_f and dsx_r bind in all *dsx* transcripts and was used to amplify fragments of different lengths depending on sex, separated by gel electrophoresis (B). RT-qPCR primers bound transcript specific and were used for quantitative reverse transcription PCR. An amino acid level alignment of the putative autoregulatory CAM domain of Tra with insect Tra orthologs is shown (D)

To identify the genes involved in the conserved part of the sex determination cascade of *A. lythri*, all transcripts of doublesex (*dsx*), transformer (*tra*), and transformer 2 (*tra2*) were extracted from the male and female transcriptomes. The in silico analysis identified three variants of *dsx* mRNA. Since a final genome assembly is not yet available for *A. lythri*, exon–intron boundaries could not be fully resolved. Nevertheless, first unpolished genome data of *A. lythri* give an indication of the size and location of the introns. A nucleotide alignment of the detected *dsx* sequences revealed three different splice variants of the *dsx* gene, spread across two of the current genome contigs (Fig. 1A). The expression analysis confirmed sex-specific differences in amount of mRNA transcripts between males and females, and the presence of three different splice variants, *dsxf1*, *dsxf2*, and *dsxm* in the females*,* of which *dsxf1* and *dsxf2* are not expressed in males (Fig. 1B). As is typical for a transcription factor, Dsx of *A. lythri* harbors the conserved N-terminal DNA binding domain (the DM motif) and the oligo dimerizing domains OD1 and OD2a/b, as reported for orthologs of other insect species (Bayrer et al. 2005). *Dsxf1*, *dsxf2*, and *dsxm* share the same start codon and result in coding sequences of 862 bp, 849 bp, and 1152 bp (307 aa, 282 aa, and 383 aa), respectively. *Dsxm* possesses the male-specific C-terminal dimerization domain (Fig. 1A, blue box), whereas *dsxf1* and *dsxf2* both carry the female-specific dimerization domain (Fig. 1A, pink box), both similar to those described for *T. castaneum* (Shukla and Nagaraju 2010). Protein sequence comparison for Dsxf1 and Dsxf2 showed 61% identity with *T. castaneum* (AFQ62106.1 and AFQ62107.1) and 77% identity with protein doublesex isoform X2 of *Diabrotica virgifera virgifera* (XP_050505026). Protein sequence comparisons between *T. castaneum* and *A. lythri* showed 53% identity for Dsxm (AFQ62105.1) and 82% identity with *Psylliodes chrysocephala* (CAH1110047.1).

For *tra*, three different splice variants were detected in the transcriptomes (Fig. 1C). Only one splice variant generates a full-length protein with 1764 bp (587 aa). Two additional variants were found, which harbor premature stop codons in their coding sequence and end after 231 bp and 492 bp (76 aa and 163 aa), respectively. The expression analysis revealed the full-length transcript as female specific, and was therefore named *traf1*, and the shortest transcript as male specific, named *tram*. *Traf1*, and the 492 bp transcript differ only in an additional exon of 69 nucleotides at the 3′ end of the 492-bp transcript containing 78% adenine, a base composition which ruled out the design of a qRT-PCR primer for a sex-specific quantification of this variant. However, this transcript was only found in the transcriptome of a female beetle and thus named *traf2*. Traf of *A. lythri* (AlyTraf) is a SR-type protein and is characterized by a putative autoregulatory domain (CAM domain) (Fig. 1D), an arginine/serine-rich domain and a proline-rich domain (Fig. 1C). Comparison with the genomic data showed that the *tra* transcripts, located on the genome contig 3382, are divided into five exons. The last 49 and 9 nucleotides of and *tram*, respectively, could not be placed on the genome, probably due to incompleteness of the latter (Fig. 1C). *Traf2* only retains the putative autoregulatory domain. AlyTraf shows only 20% identity across the entire protein sequence with the female-specific feminizer isoform of *T. castaneum* (AFQ62109.1), while only the conserved SR domains reaches up to 38% sequence identity.

*Transformer 2* (*tra2*) seems to be represented by two paralogous genes (named tra2.1 and tra2.2) and an additional pseudogene (Fig. 1E). *Tra2.1* was recovered from the transcriptomes with three different splice variants (*tra2.1_1*, *tra2.1_2*, and *tra2.1_3*). Two of these splice variants (*tra2.1_1* and *tra2.1_2*) match the splice variants found in the tra2 paralog tra2.2. The translated amino acid sequences of the paralogs are 93% identical for splice variant 1 (*tra2.1_1* vs *tra2.2_1*) and 91% identical for splice variant 2 (*tra2.1_2* vs *tra2.2_2*), respectively. All variants of *tra2* code for full-length proteins of either 666 bp (221 aa) or 678 bp (225 aa) show the conserved RNA recognition motif (RRM) domain for interaction with RNA as a splicing factor. Mapping of the different *tra2* transcripts to the unpolished genome data showed that *tra2.1* and *tra2.2* aligned to different contigs (contig 11,539 for *tra2.1* and 7799 and 9612 and 16,962 for *tra2.2*, respectively) which supports that the genes are paralogs. *Tra2.1* was mapped with 100% identity, and the three different splice variants split into six exons along the contig, of which only the last nine base pairs of transcript *tra2.1_1* could not be aligned to the genome. Mapping *tra2.1* transcripts to the genome (contig 12,683) revealed a hit without introns but multiple (41) mismatches and indels with an overall identity of 93%, which may indicate a pseudogene (Fig. 1E). The sequence with the indels and mismatches would no longer result in a functional Tra protein.

### Absence of males in eggs and larvae of HT1/HT1*

**Fig. 2:**
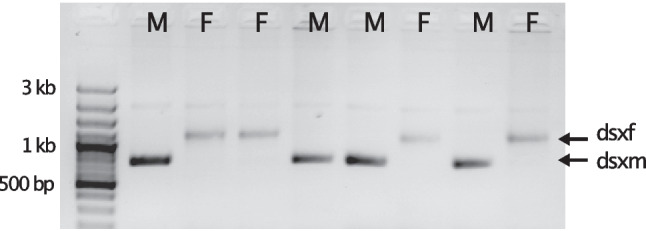
Exemplary gel electrophoresis of *dsx* RT-PCR enabling sex determination in eggs and larvae of *Altica lythri*. Here, larvae of HT2 females were screened. Male progeny (M) shows the *dsxm* splice variant, and female progeny (F) shows one or both of the *dsxf* splice variants

Our RT-PCR-based screening method for the sex-specific splice variants of doublesex could clarify at which point during development male offspring is lost in HT1/HT1* females. The progeny of HT1 and HT2 *A. lythri* females was screened with RT-PCR in the egg and larval stage with *dsx* specific primers, allowing sex to be determined by the differing length of the resulting fragments (Fig. 2). Validation of the screening method with adult male and female beetles revealed both male- and female-specific transcripts in females. In contrast, only the male-specific splice variant could be detected in adult males which agrees with the results from qRT-PCR and transcriptomes.

**Fig. 3:**
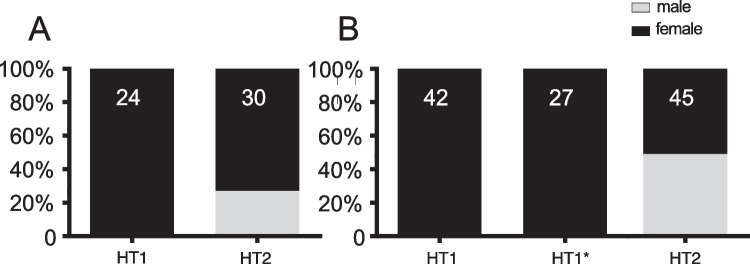
Proportion (%) of males and females in egg (A) and larval (B) stages of *Altica lythri*. Sex was determined via RT-PCR of the *dsx* splice variants for the progeny of HT1 and HT2 female beetles in eggs and of HT1, HT1*, and HT2 females in larvae. Numbers indicate total sample size for each haplotype

The progeny of HT1 (always infected with *Wolbachia* strain wLytA1) or HT1* females (uninfected) showed no male-specific *dsx* PCR fragment patterns in eggs or larvae, whereas female-specific splice variants were always present, confirming 100% female progeny (Fig. 3A). In contrast, we detected only male-specific transcripts in roughly half of the progeny of HT2 females accounting for 27% males at the egg stage (Fig. 3A) and 49% males at the larval stage (Fig. 3B).

### Expression of the sex determination genes dsx, tra, and tra 2 during development

**Fig. 4:**
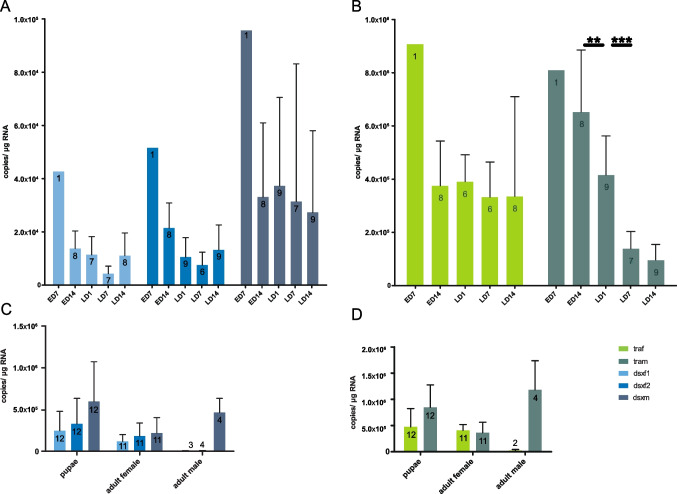
Expression profiles of *Altica lythri dsx* and *tra* transcripts in pools of 3 eggs and individual larvae (A, B) and at pupal and adult stage (C, D) analyzed by qRT-PCR (E, egg; L, larvae; D, day). Mean values with standard deviation are shown. Numbers indicate sample size. Egg day 7 was excluded from the statistical analysis due to the lack of replicates

For a better understanding of the temporal development of the sex determination pathway in *A. lythri*, we quantified the mRNA levels of the sex-determining genes (*dsx*, *tra*, and *tra2*) during *A. lythri* ontogeny from egg through larval and pupal stages to adulthood using qRT-PCR. At 18 °C, the larvae hatch about 15–17 days after oviposition, and then feed for approximately 3 to 4 weeks until they pupate. Three weeks later, the adults hatch from the pupae. The short length of the specific part of *traf2* prevented the design of a specific oligonucleotide for the analysis of its mRNA expression levels by qRT-PCR; therefore, *traf2* could not be analyzed separately from *traf1*. The results show differential expression of *dsx* and *tra* transcripts between the sexes and concordant expression of *tra2* during the ontogeny of *A. lythri*. Overall expression levels were higher in the early egg stages than in the following egg and larval development. *Tra* was overall significantly higher expressed than *dsx* (Tukey’s multiple comparison test *p* < 0.0001). The *dsxf1* and *dsxf2* transcripts showed similar expression patterns during ontogeny (Fig. 4A, C). The mRNA levels of *dsxf1* and *dsxf2* showed the common trend that expression strength tended to decrease (but not significantly) during egg (after day 7) and larval development, whereas *dsxm* remained at a rather constant level (Fig. 4A). The *dsxf* expression increased again rapidly at the last week of the larval stage, yet in pupae and adult females, the expression values were vastly higher (Fig. 4A, C). *Dsxm* showed significantly higher expression levels compared to *dsxf1* and *dsxf2* (paired *t* test *dsxf1 vs dsxm p* = 0.0034, *dsxf2 vs dsxm p* = 0.014) (Fig. 4A). In adult beetles, *dsxm* showed equally high values as *dsxf* (Fig. 4C).

The analysis of the mRNA levels of *tra* showed that both *traf* and *tram* started with similar moderate expression levels (Fig. 4B). While *tram* decreased significantly during the first two weeks of larval development (Tukey’s multiple comparison test ED14 *vs* LD1 *p* = 0.01; LD1 *vs* LD14 *p* = 0.001); *traf* remained at a rather constant level. With the beginning of the pupal stage, the expression of *tram* transcripts increased to the initial level (Fig. 4B). The highest level of all *tra* transcripts was reached by *tram* in adult males (Fig. 4D).

*A. lythri tra2* paralogs and splice variants could not be discriminated, but the use of a single pair of oligonucleotides showed a constant level of *tra2* expression over the course of development of about 10^6^ copies/μg RNA (supplementary figure S1, supplementary material online).

### Transfer of traf mRNA from mother to offspring

To gain more detailed information about the initiation of the sex determination cascade in *A*. *lythri*, we investigated whether *traf* mRNA is provided from mother to offspring to start the female sex determination cascade as was shown for *T. castaneum* (Shukla and Palli 2014).

**Fig. 5:**
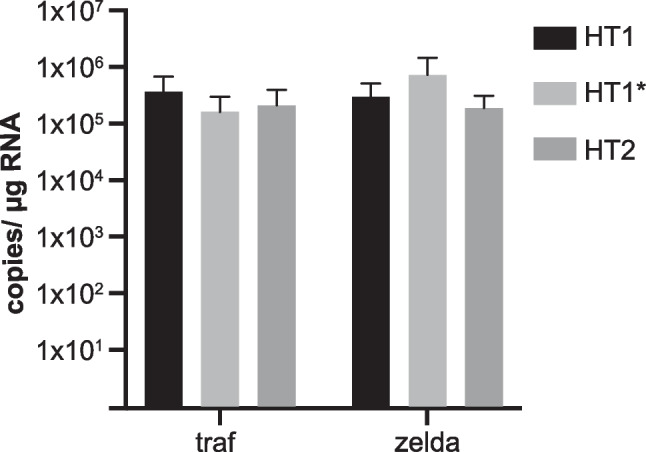
Amount of *traf* and *zelda* mRNA in 3-h-old eggs after oviposition, analyzed by RT-qPCR. Mean values with standard deviation are shown. Each bar is represented by three samples, where one sample consists of 20 pooled eggs, separated by the haplotype of the mother. No significant difference can be found between the haplotypes or the amount of *traf* versus *zelda*, thus indicating a maternal transfer of *traf* mRNA to the eggs

Indeed, mRNA of *traf* could be detected in all egg samples of *A. lythri* (pools of 20 maximally 3-h-old eggs, separated by haplotypes), with similarly high values in all haplotypes (Fig. 5). As this is too early for zygotic transcription, the mRNA must stem from maternal transmission. The well-known maternally transmitted positive control *zelda* appeared with an equally strong signal in the qRT-PCR (Fig. 5).

No statistically significant difference was found between the different haplotypes or compared to *zelda*, indicating a transmission of both *zelda* and *traf* mRNAs from mother to offspring.

## Discussion

The flea beetle *A. lythri* shows haplotype-dependent sex ratio distortion culminating in the complete lack of males in HT1/HT1* beetles. The identification of the conserved elements of the sex determination cascade and their splice variants in both sexes enabled us to trace the lack of males to the earliest time point these splice variants predict the sex of the developing offspring. As we discuss below, the initiation of the signaling pathway leading to the male phenotype must be prevented before the onset of zygotic transcription, possibly under the control of parasitic *Wolbachia* endosymbionts or due to genetic conflicts created by introgressed mitochondria.

### Sex determination cascade in *A. lythri*

**Fig. 6:**
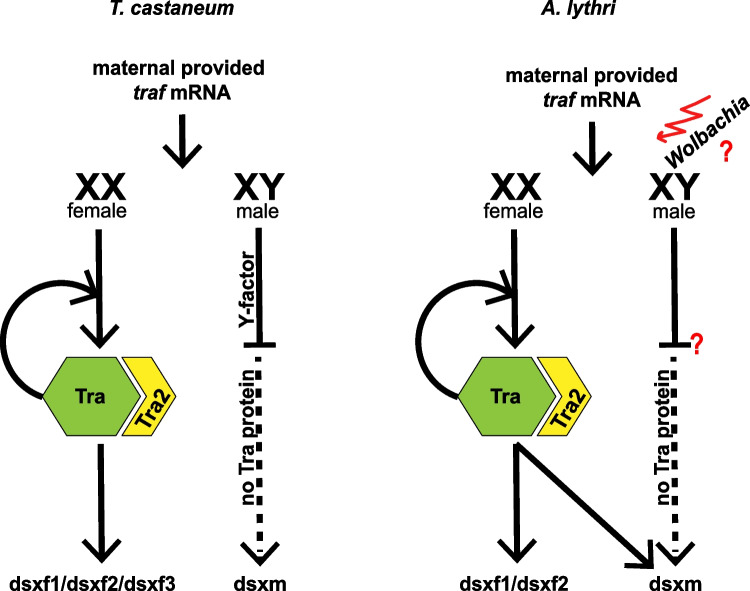
Possible scenario of the sex determination cascade in *Altica lythri* in comparison to *Tribolium castaneum*

Sex determination in insects follows a conserved regulatory cascade with transformer (Tra) as a central protein (reviewed in Verhulst et al. 2010). In females, a functional Tra protein regulates the splicing of female-specific *dsx* variants (Bopp et al. 2014; Sanchez 2008; Verhulst et al. 2010), whereas the absence of the Tra protein in males leads to default splicing and production of male-specific *dsx* variants (Cline and Meyer 1996). Tra and Dsx have different levels of evolutionary divergence that coincide with their function. Dsx, as a transcription factor regulating the expression of sex-shaping genes, harbors the very conserved oligodimerization domains OD1 and OD2, which enable the Dsx protein to interact with other proteins and DNA (Cho and Wensink 1997). Tra, as a SR-type protein building a spliceosome complex, shows a high degree of evolutionary divergence, where only the SR motifs are conserved. To form the splicing complex, 10–20% of serin-arginine (SR) dipeptides in the Tra protein are enough to maintain the functionality (Kulathinal et al. 2003). Besides the SR domain, Tra contains a putative autoregulatory domain conserved in Diptera, Hymenoptera, and Coleoptera—also found here in Traf of *A. lythri*—but not in *Drosophila* (Verhulst 2011). Once the female-specific splicing of *tra* is switched on, the continued production of Tra protein is required to maintain female-specific splicing of *tra* pre-mRNA (Pane et al. 2002; Verhulst et al. 2010). To initiate the Tra autoregulatory loop, *tra* mRNA is maternally provided in *T. castaneum* to male and female offspring. In females, the mRNA is translated into Tra protein and starts the female cascade, whereas in males, an unknown Y factor inhibits the translation of the maternally provided *tra* mRNA (Shukla and Palli 2014) (Fig. 6).

In *A. lythri*, the presence of the conserved autoregulatory domain in AlyTraf suggests a similar autoregulatory feedback loop of Traf in female sex determination (Fig. 6). This feedback loop is clearly reflected in the qRT-PCR expression analysis during ontogeny, where the amount of *traf* mRNA remained constant, corroborating its sustained expression. In contrast, the amount of *tram* mRNA decreased significantly over the egg and larval stages. As is known for *T. castaneum*, we could show with qRT-PCR in 3-h-old eggs that in *A. lythri*, *traf* mRNA is transferred from mother to offspring and presumably triggers the start of the sex determination cascade. The zygotic transcription starts in *T. castaneum* 3–6 h after oviposition (Ribeiro et al. 2017). Due to the significantly longer development time *of A. lythri* compared to *T. castaneum*, it can be assumed that zygotic transcription has not yet started in the 3-h-old eggs, and consequently that the detected mRNA must be exclusively the mRNA transferred from the mother. How and by which factor the translation of the provided *traf* mRNA is blocked in males are not yet known and requires further analyses.

In order to properly splice *dsx* in females, the Tra protein has to form a splicing complex with Tra2. In Diptera, Hymenoptera, Lepidoptera, and Coleoptera, *Tra2* does not have a sex-differentiated expression (Belote and Baker 1982; Burghardt et al. 2005; Nissen et al. 2012; Niu et al. 2005; Salvemini et al. 2003; Shukla and Palli 2013; Suzuki et al. 2012), and the same picture appears in *A. lythri* where *tra2* is constitutively expressed in both sexes at high concentrations. Tra2 also seems to be involved in larval development in *T. castaneum* (Shukla and Palli 2013), in female ovarian development in *Aedes* (Li et al. 2019) and in lipid metabolism in *Drosophila* (Mikoluk et al. 2018). In *A. lythri*, the *tra2* gene is duplicated with a strong conservation of the splice variants of the two paralogs, suggesting a recent duplication. The additional presumptive pseudogene is idiosyncratic for *A. lythri* and was not described in other insects.

### The potential influence of selfish genetic elements on female bias

Strongly distorted sex ratios as observed here in *A. lythri* may be caused by genomic conflicts over sex chromosomes or sex-determining factors (Perlman et al. 2015). Two types of selfish genetic elements are known to manipulate the sex of their hosts: cytoplasmic inherited endosymbionts (like *Wolbachia*, *Rickettsia*, *Spiroplasma*, and *Cardinium*) and selfish genetic elements on sex chromosomes (Hodson and Perlman 2019). In addition, mitochondria as cytoplasmic elements may also benefit the female sex to the expense of males (Burt and Trivers 2006; Perlman et al. 2015), as has been repeatedly shown for cytoplasmic male sterility in plants (He et al. 2021; Wang et al. 2022). Nevertheless, in animals, only negative effects on male fertility have been demonstrated and not a complete eradication of the male sex as observed in *A. lythri* HT1/HT1* (Frank and Hurst 1996; Innocenti et al. 2011; Ruiz-Pesini et al. 2000).

Of the three mtDNA haplotypes observed in *A. lythri* (HT1/HT1*, HT2, and HT3), two can be attributed to introgressive hybridization with other *Altica* species, although it still remains unclear which haplotype was originally present (Jäckel et al. 2013). *A. lythri* shows haplotype-dependent sex ratio distortion that is extreme in HT1/HT1*, with males completely absent (Jäckel 2011; Jäckel et al. 2013). Our search for male-specific *dsx* patterns across all developmental stages did not provide signs of male offspring for HT1 or HT1*, while we identified male-specific *dsx* patterns in eggs and larvae of HT2. The comparison corroborates that we would have detected males in HT1/HT1* if there were any.

Previous studies hypothesized that sex ratio distortion in *Altica* may be due to infection with *Wolbachia* bacteria (Jäckel et al. 2013). To increase its own transmission, *Wolbachia* has evolved three main mechanisms to increase the number of females in a population: male killing, feminization of genetic males, and induction of parthenogenesis (Engelstädter and Hurst 2009). HT1 females are pervasively infected with the *Wolbachia* strain wLytA1(Jäckel 2011; Jäckel et al. 2013); from this perspective, it seemed likely that wLytA1 causes the female bias. The absence of male progeny in eggs and larvae of HT1 and HT1* females allows us to exclude *Wolbachia* induced male-killing as a sex distortion mechanism. However, feminization by *Wolbachia* cannot yet be fully excluded because we lack genetic markers for the sex chromosomes. The current assembly status of the draft genome is not yet sufficient for such an analysis but rather first requires further improvements. The current *tra* and *dsx* markers are based on phenotypic transcriptional differences rather than underlying genetic differences. If *Wolbachia* manipulated the sex determination cascade, feminization of genetic males could still occur (Fig. 6) as was observed in the butterfly *Eurema mandarina*, where *Wolbachia* is altering the splicing of *doublesex* (Narita et al. 2007).

Nevertheless, the observation that the uninfected HT1* females also exclusively produced female offspring argues against *Wolbachia* as a cause of sex ratio distortion in *A. lythri*. This haplotype represents a minor sequence variant of HT1, but females carrying it are usually not infected with *Wolbachia*. However, we cannot yet exclude a horizontal transfer of *Wolbachia* sex distortion genes into the nuclear genome of HT1* *A. lythri*. This would mean that even without positive evidence of acute *Wolbachia* infection, genes exported from *Wolbachia* to the genome could still have an effect on sex ratio. Gene transfer from *Wolbachia* into invertebrate genomes or even sex chromosomes has been repeatedly observed (Dunning Hotopp et al. 2007; Kondo et al. 2002; Nikoh et al. 2008). *Wolbachia* genes were recently detected in the genome of the “distorter” booklouse (*Liposcelis* sp., Psocodea) but not in the normally reproducing type, making these horizontally transferred genes possible candidates causing the sex ratio distortion in this species (Hamilton et al. 2018).

Further investigations into the fascinating system of the hybridogenetic *A. lythri* beetles will shed light on how genetic conflicts shape sex determination in this species. Starting from the foundation laid in this paper, we may now embark on in-depth investigations to clarify whether the primary sex-determining signal is manipulated by selfish genetic elements or genomic conflicts that resulted from interspecies hybridization.

## Supplementary Information

Below is the link to the electronic supplementary material.

Supplementary file1 (DOCX 76.9 KB)

## Data Availability

The data that support the findings of this study are available in ENA (European nucleotide archive) as BioProject Acc.No. PRJEB50615. The individual sex determination gene sequences of *A. lythri* can be found under accession numbers OX442477-OX442487. Raw long reads and details of the sequencing of the *A. lythri *genome used in this study are available in NCBI (Bioproject: PRJNA947484).
