# Supplementary material for: When does the female bias arise? Insights from the sex determination cascade of a flea beetle with a strongly skewed sex ratio
Source: Funct Integr Genomics. 2023 Mar 31;23(2):112. doi: 10.1007/s10142-023-01023-1 (PMC10066108; doi:10.1007/s10142-023-01023-1)
Supplement: Supplementary file 1 — Supplementary file1 (DOCX 76.9 KB) [file 10142_2023_1023_MOESM1_ESM.docx]

**Supplementary material legend:**

**Fig. S1:**

Expression profiles of *Altica lythri* *tra2* transcripts analyzed by qRT-PCR at different timepoints during their development. (Abbr. E = egg, L= larvae, D = day). Mean values with standard deviation are shown. Numbers indicate sample sizes.

Table S1: Oligonucleotides used for RT-PCR, qRT-PCR and One-Step RT-PCR.

| Gene name | Primer-label | 5´-3´sequence |
| --- | --- | --- |
| doublesex  (dsx) | dsx_r | GCGTTACTCTTGATTCAGC |
|  | dsx_f | ACTTCAGAACGACAACGAG |
|  | dsx 5'UTR for | AATGATTGCCCTGCTTGCC |
|  | dsx 3'UTR rev | GGCAATTTCTAATGTAAATGC |
|  | RT-qPCR dsx m rev | CGATAGTTTGTCCTTCATCTATCCTTCG |
|  | RT-qPCR dsx f1 rev | CTTCGGATGCGTGGAATTCC |
|  | RT-qPCR dsx f2 rev | ATCATCCATACTGACGCGTGG |
|  | RT-qPCR dsx for | GAACGATTTAAGTATCCGTGGGA |
| transformer  (tra) | RT-qPCR tra i5 for | GGCTCCTGCACCCATTGTAAGG |
|  | RT-qPCR tra i5 rev | CATTTACAATTCCTCAGTCCTCTTCTC |
|  | RT-qPCR tra i1 for | GTTCACATTAAAAAGGGCAACAATC |
|  | RT-qPCR tra i1 rev | CTAGGAACTACATTACGTTGCCG |
| transformer 2.1 (tra2) | RT-qPCR tra2 for | GGAAAACCGACATATAAATATTCCGAC |
|  | RT-qPCR tra2 rev | TTCTGCTGCTGTAGTATGGAGAAG |
| Cytochrome oxidase (COI) | S1634 | ATTGGAGATGAYCAAATTTATAYYGT |
|  | A2969_Alyt | AGTCAGAGTATGAGTGTTCAG |
| *Wolbachia* surface protein (wsp) | wsp_for | TGGTCCAATAAGTGATGAAGAAAC |
|  | wsp rev | AAAATTAAACGCTACTCCAG |
| *Wolbachia* Cytochrome c oxidase subunit I (coxA) | CoxA_F1 | TTGGRGCRATYAACTTTATAG |
|  | CoxA_R1 | CTAAAGACTTTKACRCCAGT |
| *Wolbachia* filamentation temperature-sensitive protein Z (ftsZ) | FtsZ_GF1 | ATYATGGARCATATAAARGATAG |
|  | FtsZ_GR1 | TCRAGYAATGGATTRGATAT |
| zelda  (zld) | Aly_zld_qPCR_for | GTGCTGATCAACGAATCGCC |
|  | Aly_zld_qPCR_rev | GCCTTATGCCTGCGATACCT |
